# Joint Metabolomics and Transcriptomics Reveal Rewired Glycerophospholipid and Arginine Metabolism as Components of BRCA1-Induced Metabolic Reprogramming in Breast Cancer Cells

**DOI:** 10.3390/metabo15080534

**Published:** 2025-08-07

**Authors:** Thomas Lucaora, Daniel Morvan

**Affiliations:** 1 Centrale Nantes, Rue De La Noë, 44321 Nantes Cedex 3, France; thomas.lucaora@gmail.com; 2 Department of Biophysics, Faculty of Medicine, UCA University, Boulevard François Mitterrand, 63001 Clermont-Ferrand, France; 3 Comprehensive Cancer Centre Jean Perrin, Rue Montalembert, 63011 Clermont-Ferrand, France

**Keywords:** breast cancer, BRCA1, metabolic reprogramming, joint metabolomics/transcriptomics, two-way orthogonal partial least squares, pathway analysis

## Abstract

**Background/Objectives**: The breast cancer susceptibility gene 1 (*BRCA1*) is a tumor suppressor gene whose mutations are associated with increased susceptibility to develop breast or ovarian cancer. *BRCA1* mainly exerts its protective effects through DNA double-strand break repair. Although not itself a transcriptional factor, BRCA1, through its multiple protein interaction domains, exerts transcriptional coregulation. In addition, BRCA1 expression alters cellular metabolism including inhibition of de novo fatty acid synthesis, changes in cellular bioenergetics, and activation of antioxidant defenses. Some of these actions may contribute to its global oncosuppressive effects. However, the breadth of metabolic pathways reprogrammed by BRCA1 is not fully elucidated. **Methods**: Breast cancer cells expressing BRCA1 were investigated by multiplatform metabolomics, metabolism-related transcriptomics, and joint metabolomics/transcriptomics data processing techniques, namely two-way orthogonal partial least squares and pathway analysis. **Results**: Joint analyses revealed the most important metabolites, genes, and pathways of metabolic reprogramming in BRCA1-expressing breast cancer cells. The breadth of metabolic reprogramming included fatty acid synthesis, bioenergetics, HIF-1 signaling pathway, antioxidation, nucleic acid synthesis, and other pathways. Among them, rewiring of glycerophospholipid (including phosphatidylcholine, -serine and -inositol) metabolism and increased arginine metabolism have not been reported yet. **Conclusions**: Rewired glycerophospholipid and arginine metabolism were identified as components of BRCA1-induced metabolic reprogramming in breast cancer cells. The study helps to identify metabolites that are candidate biomarkers of the *BRCA1* genotype and metabolic pathways that can be exploited in targeted therapies.

## 1. Introduction

Loss of expression of the tumor suppressor gene breast cancer susceptibility gene 1 (*BRCA1*) by mutation, methylation, or other mechanisms is at the origin of an increased risk of developing breast or ovary cancer. The BRCA1 protein has multiple functions in cells, including DNA double-strand break repair mostly by homologous recombination, cell cycle regulation, protein ubiquitination, and chromatin remodeling. BRCA1 interacts with multiple cellular proteins including the histone deacetylase complex, zinc finger proteins, the estrogen and androgen receptors, and transcription factors (p53, c-Myc). Although it is not itself a transcription factor, BRCA1 has been shown to intervene as a coregulator of transcription [1]. More recently, BRCA1 was found to impact cellular metabolism. BRCA1 inhibits de novo fatty acid (FA) synthesis by direct interaction with acetyl-CoA carboxylase 1 (ACC1), thus stabilizing the enzyme in its phosphorylated and inactive form [2]. It enhances the activity of the antioxidant response of the transcription factor nuclear factor erythroid-derived 2-like 2 (Nrf2), and reduces intracellular reactive oxygen species (ROS) [3,4,5]. BRCA1 expression was shown to decrease glucose consumption and increase oxygen consumption of breast cancer cells [6], as well as increase mitochondrial respiration and ATP content of ovarian cancer cells [7,8]. Hypoxia-inducible-factor 1 (HIF-1) alpha which is a major regulator of bioenergetics was more frequently overexpressed in *BRCA1*-mutated breast cancers [9]. Mechanistically, BRCA1 was shown to regulate HIF-1 stability [10]. Recently, it was reported that BRCA1 associated with a zinc finger protein, which bound to the promoter of *GOT2* and repressed the gene [11]. Also, *BRCA1* mRNA expression was shown to inversely correlate with mRNA and protein expression of nicotinamide N-methyltransferase (NNMT), an emerging regulator of metabolism [8]. Some metabolic functions of BRCA1 could play a role in its oncosuppressive properties. As a matter of fact, ACC1 inhibition unrelated to BRCA1, and inhibition of other enzymes initiating de novo FA synthesis, exhibit antitumor effects [12,13,14].

Although global transcriptomics of BRCA1 expression have deserved a number of investigations, metabolomics studies of BRCA1 expression as well as metabolism-centered transcriptomics are few [6,15]. Metabolomics was used to show bioenergetics changes in BRCA1-expressing breast cancer cells [6]. Another metabolomics study identified adenine (Ade), as well as other purine base derivatives in breast cancer cell lines and human plasma, as biomarkers of the *BRCA1* genotype [15]. However, no metabolomics study evaluated the extent of metabolic pathways related to BRCA1 expression. To this aim, we performed a joint metabolomics/transcriptomics investigation of breast cancer cells expressing BRCA1.

First, using intact cell NMR metabolomics, we showed decreased cellular FA content in BRCA1-expressing breast cancer cells. Then, multiplatform metabolomics and transcriptomics data were acquired and analyzed using joint metabolomics/transcriptomics data processing techniques as an improvement in comparison with univariate statistical analysis. The breadth of altered metabolic pathways of BRCA1-expressing breast cancer cells included downregulation of glycolysis, inhibition of HIF-1 signaling pathway, upregulation of phosphatidylserine (PtS) synthesis from base exchange with phosphatidylcholine (PtC) and other glycerophospholipid metabolism alterations, upregulation of glutathione (GSH) metabolism, decreased de novo nucleotide synthesis, and increase in arginine (Arg) metabolism. Among altered pathways, rewired glycerophospholipid and Arg metabolism have not been reported yet. Also, we show that the fate of serine is central to BRCA1-induced metabolic reprogramming and governed by transcription of few canonical genes of metabolism.

The study helps to identify metabolites that are candidate biomarkers of the *BRCA1* genotype, understand global oncosuppressive effects of *BRCA1*, and identify metabolic vulnerabilities that can be exploited in targeted therapies.

## 2. Materials and Methods

### 2.1. Cell Culture and Transfection

The SUM1315MO2 human breast cancer cell line was obtained from Asterand (Detroit, MI, USA). The SUM1315MO2 cell line harbors the 185delAG *BRCA1* mutation and is classified as a triple-negative breast cancer (TNBC). Clinically, TNBCs are characterized by their aggressiveness and lack of targeted therapy. The procedure to obtain stable transfection of SUM1315MO2 breast cancer cells with wild-type *BRCA1* has been previously described [16]. Briefly, cells were grown in Ham’s F12 medium at 37 °C in a humidified atmosphere containing 5% CO_2_. SUM1315MO2 cells were transfected with either a pLXSN plasmid containing the full-length *BRCA1* cDNA using FuGENE 6 transfection reagent (Promega, Madison, WI, USA) or a pLXSN empty vector. Clones were selected in 721.5 μM G418 disulfate salt solution (Sigma Aldrich, St.Louis, MO, USA). The BRCA1-expressing cell line (SUM1315-BRCA1) and the control cell line (SUM1315-CT) were grown in a culture medium containing 721.5 mM G418.

### 2.2. Intact Cell NMR Spectroscopy

Cultured cells were harvested from plates by trypsinization and rinsed with phosphate-buffered saline–D_2_O. Pellets were stored at −80 °C until NMR spectroscopy analysis. NMR spectroscopy was performed on a small-bore 500 MHz Bruker Avance spectrometer (Bruker Biospin, Rheinstetten, Germany), equipped with a high-resolution magic angle spinning probe. Intact cell pellets were set into 4 mm diameter, 50 μL zirconium oxide rotor tubes with 2 drops of D_2_O to lock the spectrometer. Rotors were spun at 4 kHz and cooled at 4 °C using the BCU-05 temperature unit. Acquisition and minimal data processing were performed using the Topspin Version 2.1 software (Bruker Biospin). One-dimensional ^1^H-NMR spectra were obtained using a nuclear Overhauser enhancement spectroscopy sequence with low-power water signal presaturation (NOESYPR) during both the 3.8 s relaxation delay and the 100 ms mixing time of the sequence. The acquisition spectral width was 12 ppm, with 16,384 complex data points and 32 transients. After Fourier transformation, a baseline correction was applied using a spline function and spectra were transferred to the Excel software (Microsoft) for signal quantification according to a validated procedure [17] and to the SIMCA 14 software for multivariate analysis.

### 2.3. Metabolite Extraction

Metabolite extraction was performed in an ice-cold environment. Cells were suspended in 2 mL of methanol/chloroform (2:1, *v*/*v*) and ultrasonicated for 1 min. Then 500 μL of purified water/chloroform (1:1, *v*/*v*) were added and the organic and aqueous phases were separated by centrifugation (1500 g, 20 min, 4 °C). Both phases were evaporated under argon flux, lyophilized, and stored at –80 °C.

### 2.4. Aqueous Phase NMR Spectroscopy

The aqueous phase was analyzed in parallel by ^1^H-NMR Spectroscopy and Liquid Chromatography–Mass Spectrometry (LCMS) using the Metabolic Profiler dual analyzer (Bruker Daltonik, Bremen, Germany). Samples were prepared using a Gilson 215 liquid handler (Gilson Medical Electronics, Villiers-le-Bel, France). The dried extracts were solubilized in a solution containing 0.15 M phosphate buffer (pH 7.2), 5 mM sodium trimethylsilyl[2,2,3,3-^2^H_4_]propionate, and 2 mM NaN_3_ in D_2_O.

1D ^1^H-NMR spectra were obtained using a NOESYPR pulse sequence incorporating a z-axis spoiler gradient at 27 °C. The sequence involved a 4 s relaxation delay and a 10 ms mixing time. For each sample, 512 transients were collected into 64k data points over a spectral width of 20 ppm. After Fourier transformation, a baseline correction was applied, and spectra were transferred to the Excel software (Microsoft) for signal quantification in reference to the standard signal at 0 ppm and to the SIMCA 14 software for multivariate analysis. Signals were assigned to metabolites using the Human Metabolome Database (HMDB).

### 2.5. Aqueous Phase Liquid Chromatography–Mass Spectrometry

LCMS spectra of the aqueous phase were obtained using a microTOF ESI-TOF mass spectrometer (Bruker Daltonik, Bremen, Germany) coupled to a fast LC system (1200 Series, Agilent Technologies, Waldbronn, Germany) and controlled by the HyStar software (Bruker Daltonik). A mobile phase system consisting of water and acetonitrile (each containing 0.1% formic acid) was used, and separation was performed with an RP-18 column (Zorbax Eclipse XDB-C18 4.6×150 mm, 3.5 μm particle size, Agilent). Data sets were acquired in positive electrospray ionization mode in a scan range from 50 to 800 *m*/*z* at a sampling rate of 1 Hz. Sodium formate solution was used for calibration and injected at the beginning of each chromatographic run. Quality control samples and blank runs were interspersed between the samples. MS raw data were converted into NetCDF files using the Compass 1.3 sofware (Bruker Daltonik). Subsequent data processing was performed using XCMS (https://metlin.scripps.edu, accessed on 5 June 2025) including retention time alignment, matched filtration, peak detection, and peak matching. Then peaks were integrated. Metabolites were identified from the HMDB. For metabolite assignment, *m*/*z* accuracy was below 10^−5^.

### 2.6. Organic-Phase High-Performance Thin-Layer Chromatography

A volume of 40 mL of cellular lipid extract in chloroform was used for each sample. Lipids were separated by High-Performance Thin-Layer Chromatography (HPTLC) on a silica gel plate (Merck, Darmstadt, Germany). HPTLC plates were used after prewashing with a mixture of chloroform–methanol (1:1, *v*/*v*) followed by heating at 110 °C for 10 min. Lipid extracts were applied under a flow of nitrogen on the HPTLC plate using a Linomat IV apparatus (CAMAG, Muttenz, Switzerland) and separated using the following sequential development system: development to half final distance in methyl acetate–chloroform–propanol–methanol–0.25% KCl in water (25:25:25:10:9, *v*/*v*) followed by full development in hexane-diethylether-acetic acid (80:20:2, *v*/*v*). Quantification was performed after staining (10% CuSO_4_ *w*/*v* in 8% H_3_PO_4_ (*v*/*v*)) and charring at 160 °C. Then plates were scanned, and quantification was obtained using density measurements with the SIGMA scan pro 5.0 software.

### 2.7. RNA Extraction and Quantitative RT-PCR

Total RNAs were extracted from cell cultures using RNA PLUS reagent (Qiagen, Courtabeuf, France) according to the manufacturer’s protocol. The quality of RNAs was checked using the 2100 BioAnalyzer (Agilent Technologies). Five micrograms of RNA was then reverse-transcribed using the First-strand cDNA synthesis kit (GE Healthcare, Little Chalfont, UK). TaqMan low-density arrays (Applied Biosystems, Villebon-sur-Yvette, France) were manufactured according to a custom-defined gene panel. Low-density arrays are 384-well microfluidic cards preloaded with sets of primers and specific probes (Applied Biosystems). Transcripts were quantified using an ABI Prism 7700 thermal cycler (Applied Biosystems). Mean threshold cycles (Ct) were corrected for internal control gene (*ACTB*, *18S*) Ct to give ΔCt. Then relative quantification was obtained using the comparative threshold cycle method with ΔΔCt = ΔCt(SUM1315-BRCA1) − ΔCt (SUM1315-CT) and fold variation = 2^(−ΔΔCt)^.

The metabolism-related gene panel was elaborated from the collection of metabolic maps of the Kyoto Encyclopedia of Genes and Genomes (KEGG) database (https://www.genome.jp/kegg/pathway.html, accessed on 5 June 2025), namely the Carbohydrate, Energy, Lipid, Nucleotide, and Amino Acid pathway maps, and the expression of 90 genes involved in metabolism was quantified.

### 2.8. Univariate Statistics

Quantified metabolites and transcripts of SUM1315-BRCA1 (*n* = 10) and SUM1315-CT (*n* = 8) cells were compared using the bilateral Student’s *t*-test with a *p* value threshold of 0.05. Fold variations were calculated for the SUM1315-BRCA1 cell line in reference to the average value of variables in the SUM1315-CT cell line.

### 2.9. OPLS Data Processing of NMR Spectra

The same procedure was used for intact cell and aqueous-phase NMR spectra. Full-resolution spectra were imported into the SIMCA 14 software (Umetrics, Uppsala, Sweden) and submitted to orthogonal partial least squares (OPLS) data processing. OPLS generates a single descriptive and predictive model after fractionating the variance of data (X data) into 3 parts (predictive of the phenotype, orthogonal to the phenotype Y and residual).

To prevent overfitting of the model, a cross-validation process was applied to decide whether the component (the predictive or orthogonal ones) was significant or not. The overall quality of the model was assessed by the parameters R2X, Q2, and cross-validated (CV)-ANOVA. The former two designate the percentage of explained variance of data (total variance = 1) and the CV variance of data, respectively. A good and statistically significant model is obtained with R2X and Q2 > 0.5 and *p* value < 0.05 at CV-ANOVA. OPLS provided the following results: (i) t[1] and t0[1] scores, the projection of individual samples on the predictive and first orthogonal components; (ii) *p*(Ctr)[1] loadings, the contribution of variables (spectral data points) to the predictive component; (iii) *p*(corr)[1] correlation coefficients that gave the statistical proximity of a variable to the model.

### 2.10. O2PLS of Joint Metabolites/Transcripts

O2PLS was applied to two joint data sets referred as X = transcripts (*n* = 90) and Y = metabolites (*n* = 69), according to a procedure detailed in [18]. O2PLS decomposes the variance of data in X/Y joint (correlated), unique to X, unique to Y and residual parts. The unique to X part is the variation in X that is orthogonal (unrelated) to Y and reciprocally for the unique to Y part. The metabolite set combined measurements from three techniques (aqueous phase NMR spectroscopy, aqueous phase LCMS, organic phase HTPLC). O2PLS was performed using the SIMCA 14 software.

All data were scaled in the unit variance mode. To prevent overfitting of the model, a cross-validation process was automatically applied to define whether a component was significant or not. The overall quality of the model was assessed by R2X, Q2, and R2Y. O2PLS provided the following results: (i) t[1] and t[2] scores, the projection of individual samples on the predictive components; (ii) *p*(corr)[1] and *p*(corr)[2] correlation coefficients with the predictive components. Statistical significance of *p*(corr)[1] loadings was tested using CV-ANOVA at *p* = 0.05.

### 2.11. Pathway Analysis

Identifying enriched pathways is a common approach to biological interpretation using large-scale genomic data. MetaboAnalyst is a web-based tool suite (https://www.metaboanalyst.ca, accessed on 5 June 2025) adapted to perform metabolomics data analysis, visualization, and functional interpretation, but also transcriptomics and joint metabolomics/transcriptomics data analysis and interpretation. MetaboAnalyst was used to identify metabolic/molecular pathways from the KEGG database updated in December 2024.

The software provided overrepresentation analysis (ORA) of metabolic pathways based on the comparison of total number of molecules (enzymes/metabolites) of a pathway, number of molecules expected at random, and number of differentially varying molecules at univariate statistics. The enrichment (ratio of probed to total molecules) was tested for statistical significance using the hypergeometric test. To correct it for multiple statistical comparisons, the *p* value was adjusted using the Holm method. A pathway impact score was calculated based on topological analysis and quantified the fact that differentially varying molecules were either peripheral (low or null score) or central (high score) in the pathway. Metabolite and gene annotations were matched to KEGG identifiers. The human library was selected. The analysis was performed using metabolites only, genes only, and joint metabolites and genes.

## 3. Results

### 3.1. SUM1315-BRCA1 Intact Cells Exhibit Reduction in Intracellular Fatty Acid Levels

Due to the short representation of FA derivatives in our metabolite data set of Section 3.3 (free FA only), we performed ^1^H-NMR spectra of intact cells as an independent experiment to obtain additional information on FAs.

**Figure 1:**
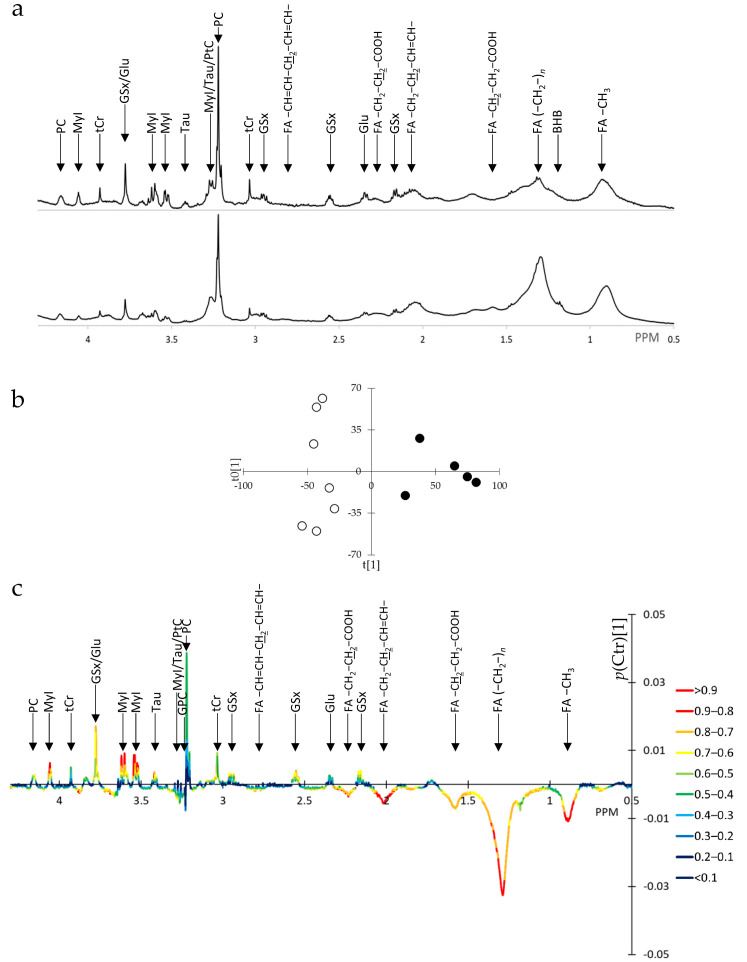
OPLS of ^1^H-NMR spectra of intact cells of SUM1315-BRCA1 vs. SUM1315-CT breast cancer cells. (**a**) Typical ^1^H-NMR spectra in the area 0.5 to 4.3 ppm; top, SUM1315-BRCA1; bottom, SUM1315-CT. (**b**) OPLS scores plot of SUM1315-BRCA1 (black dots, *n* = 7) vs. SUM1315-CT (white dots, *n* = 5). (**c**) *p*(Ctr)[1] loadings plot colored according to abs(*p*(corr)[1]) (right panel). For abbreviations and assignments, see Table S1. Underlined, protons involved in the resonance.

Typical intact cell ^1^H-NMR spectra are displayed in Figure 1a. OPLS of intact cell spectra (SUM1315-BRCA1, *n* = 7, vs. SUM1315-CT, *n* = 5) yielded a single predictive component and 2 orthogonal components with R2X = 0.782, Q2 = 0.786, and *p* < 0.05 at CV-ANOVA, thus a very good model. The predictive component completely separated samples expressing or not expressing BRCA1 (Figure 1b). The predictive component in the form of *p*(Ctr)[1] is displayed in Figure 1c and colored according to abs(*p*(corr)[1]). Signals important in the prediction with high *p*(corr)[1] values were FA signals at 0.9, 1.30, and 2.05 ppm, total glutathione (GSx) at 2.55, 2.96, and 3.77 ppm, total creatine (tCr) at 3.035 ppm, myoinositol (MyI) at 3.53, 3.62, and 4.05 ppm, and taurine (Tau) at 3.43 ppm. FA signals were decreased whereas GSx, tCr, and MyI were increased.

Then, signals of intact cell ^1^H-NMR spectra were quantified. FA resonances, namely -CH_3_ at 0.9 ppm, (-CH_2_-)*_n_* at 1.3 ppm, -CH_2_-CH_2_-COOH at 1.6 ppm, and -CH_2_-CH_2_-CH=CH- at 2.05 ppm were significantly reduced in SUM1315-BRCA1 cells: 0.70 ± 0.07 vs. 1 ± 0.22, *p* < 0.01; 0.33 ± 0.14 vs. 1 ± 0.48, *p* < 0.01; 0.28 ± 0.18 vs. 1 ± 0.14, *p* < 0.05; 0.47 ± 0.07 vs. 1 ± 0.38, *p* < 0.01; *n* = 7 vs. *n* = 5, SUM1315-BRCA1 vs. SUM1315-CT, respectively. The ratio of (-CH_2_-)*_n_* to -CH_3_ resonances was decreased in BRCA1-expressing cells (1.49 ± 0.44 vs. 0.73 ± 0.23, *p* < 0.01), suggesting that these cells coped with decreased capability to synthesize FA by shortening FA chain length. Other metabolic changes included a highly significant increase in free MyI, easily measured from its signal at 4.05 ppm: 2.61 ± 0.28 vs. 1 ± 0.55, *p* < 0.0001, *n* = 7 vs. *n* = 5, SUM1315-BRCA1 vs. SUM1315-CT, and increases in Tau (x3.18, *p* < 0.003), GSx (x1.39, *p* = 0.039), glutamic acid (Glu, x2.1, *p* = 0.003), and tCr (x1.32, *p* = 0.044).

### 3.2. SUM1315-BRCA1 Water-Soluble Extracts Exhibit Numerous Metabolite Alterations

As a matter of comparison with ^1^H-NMR spectra of intact cells, we provide ^1^H-NMR spectra of extracted cells. Both spectrum types inform on the absolute concentration of several metabolites, especially those that were candidate biomarkers of the *BRCA1* genotype such as MyI.

**Figure 2:**
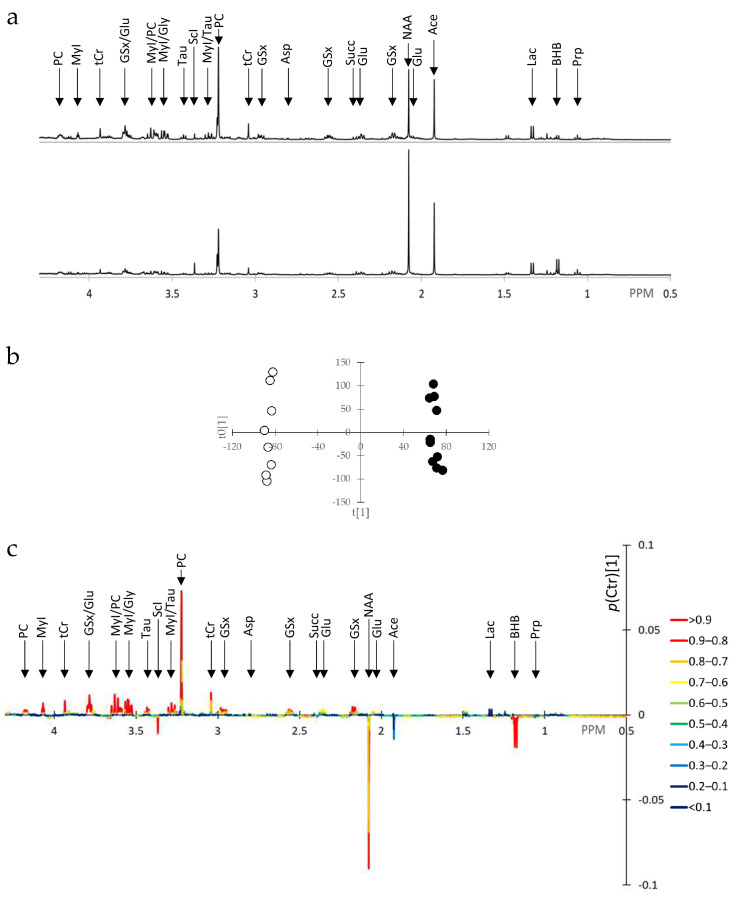
OPLS of ^1^H-NMR spectra of cell extracts of SUM1315-BRCA1 vs. SUM1315-CT breast cancer cells. (**a**) Typical ^1^H-NMR spectrum in the area 0.5 to 4.3 ppm; top, SUM1315-BRCA1; bottom, SUM1315-CT. (**b**) OPLS scores plot of SUM1315-BRCA1 (black dots, *n* = 10) vs. SUM1315-CT (white dots, *n* = 8). (**c**) *p*(Ctr)[1] loadings plot colored according to abs(*p*(corr)[1]) (right panel). For abbreviations and assignments, see Table S1.

Typical extracted cell NMR spectra are displayed in Figure 2a. OPLS of extracted cell spectra yielded a single predictive component and 2 orthogonal components with R2X = 0.539, Q2 = 0.914, and *p* < 0.001 at CV-ANOVA, thus a very good model. The predictive component completely separated samples according to BRCA1 expression (Figure 2b). The predictive component in the form of *p*(Ctr)[1] is displayed in Figure 2c, colored according to abs(*p*(corr)[1]). Signals important in the prediction with high *p*(corr)[1] value were as follows: 3-hydroxybutyric acid (BHB) at 1.18 ppm; N-acetyl-L-aspartic acid (NAA) at 2.07 ppm; GSx at 2.16, 2.55, 2.96, and 3.77 ppm; tCr at 3.035 ppm and 3.93 ppm; phosphorylcholine (PC) at 3.22, 3.62, and 4.17 ppm; MyI at 3.25, 3.53, 3.62, and 4.05 ppm; scylloinositol (ScI) at 3.36 ppm; Tau at 3.27 and 3.43 ppm.

### 3.3. Joint Metabolite/Transcript Analysis Using O2PLS Identifies Most Important Metabolites and Genes of Metabolic Reprogramming

**Figure 3:**
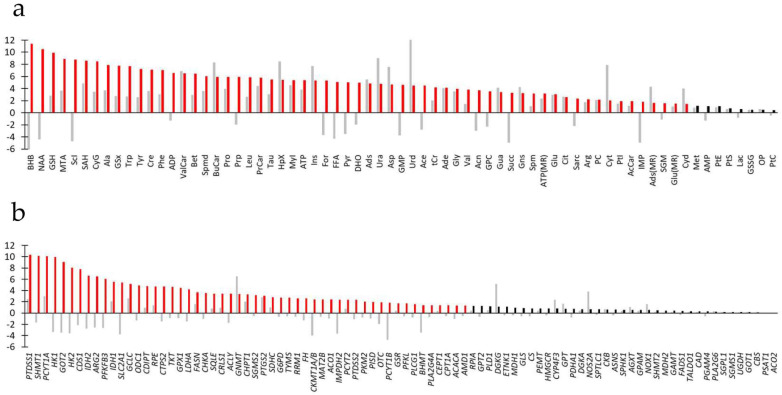
Metabolites and transcripts in BRCA1-expressing breast cancer cells ranked according to statistical significance at *t*-test (*p* value converted to −log10(*p*)). (**a**) Metabolites. (**b**) Transcripts. Gray bars: log10 transformation of fold variation showing increased or decreased variation. Black and red bars: −log10(*p*). Red: statistically significant at *t*-test. For abbreviations and symbols, see Tables S1 and S2.

Quantified metabolites (*n* = 69) from multiple platforms (NMR spectroscopy, LCMS and HTPLC of extracted cells) are given in Figure 3a and Table S1. A few metabolites or parent metabolites were measured by both NMR spectroscopy and LCMS. In all cases, metabolite pairs varied the same way (e.g., GSx and GSH), thus providing some between-technique quality control. Quantified transcripts (*n* = 90) are given in Figure 3b and Table S2. Data from all platforms were normalized to the SUM1315-CT group.

**Figure 4:**
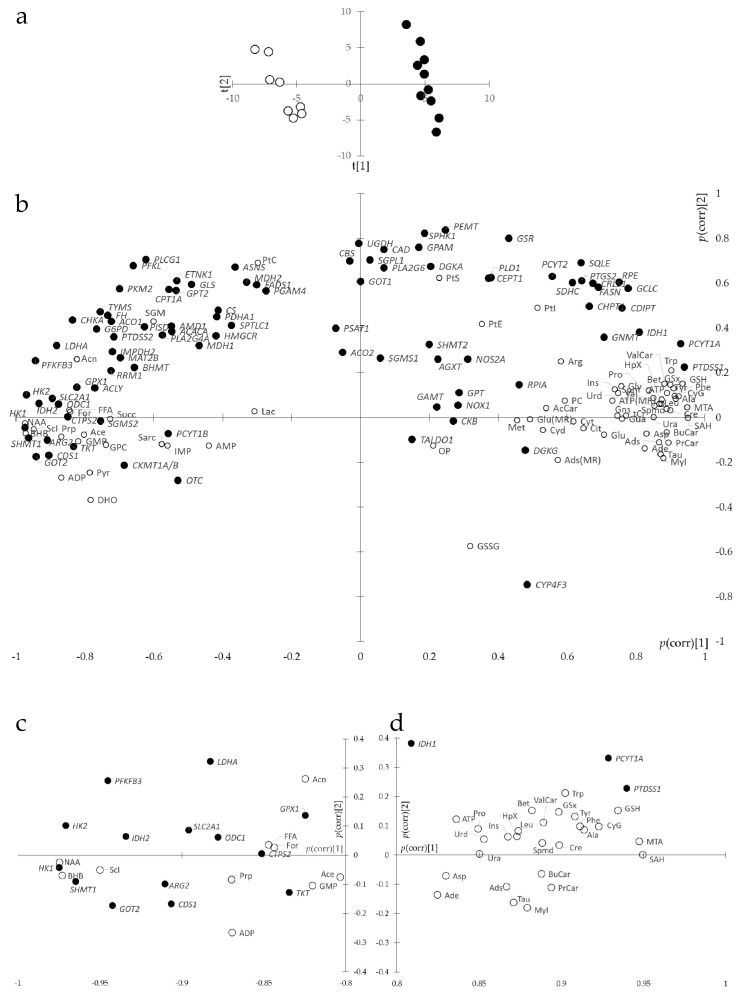
O2PLS of joint metabolites and transcripts of SUM1315-BRCA1 vs. SUM1315-CT breast cancer cells. (**a**) Scores plot of SUM1315-BRCA1 (black dots, *n* = 10) vs. SUM1315-CT (white dots, *n* = 8). (**b**) Loadings plot *p*(corr)[2] vs. *p*(corr)[1] showing metabolites (white dots) and transcripts (black dots). (**c**,**d**) Loadings plot enlargement at both extremities of the first component. For abbreviations and symbols, see Tables S1 and S2.

Given the large statistical significance at univariate statistics (*t*-test) of both metabolites (61 of 69 metabolites) and transcripts (53 of 90 transcripts), we sought for information reduction and improved ranking. O2PLS was appropriate for this aim since it enabled the combination of two different sets of data while accounting for correlations between variables that could bias univariate statistics and removing structured noise. O2PLS yielded two predictive components, two orthogonal components in X and two orthogonal components in Y, with R2X = 0.784, Q2 = 0.508, and R2Y = 0.827, thus a very good model. The first predictive component completely separated samples according to BRCA1 expression (Figure 4a). The second predictive component, representing 6% of the total predictive variance, described a common trend of breast cancer cells unrelated to BRCA1 expression. The loadings plot of O2PLS gave the correlation of metabolites and transcripts with the components (Figure 4b) so that metabolites and transcripts to the right of the loadings plot positively correlated with BRCA1 expression and those to the left negatively. Statistical significance of loadings at *p* = 0.05 was obtained by CV-ANOVA, corresponding to the threshold abs(*p*(corr)[1]) = 0.824.

**Figure 5:**
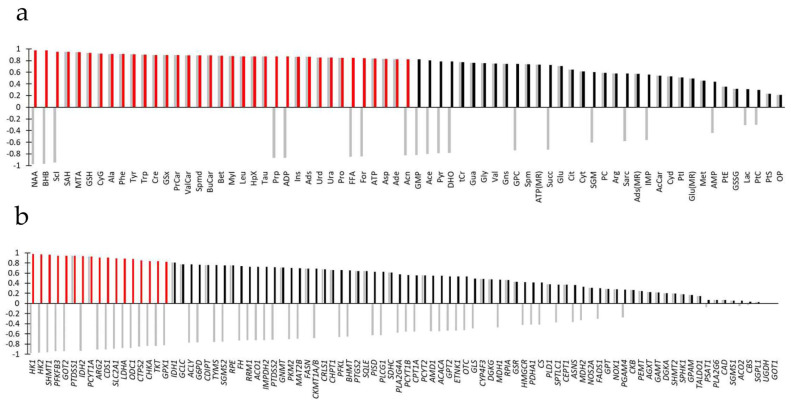
Metabolites and transcripts in BRCA1-expressing breast cancer cells ranked according to abs(*p*(corr)[1]). (**a**) Metabolites. (**b**) Transcripts. Gray bars: Signed *p*(corr)[1] showing increased or decreased variation. Black and red bars: abs(*p*(corr)[1]). Red: Statistically significant at CV-ANOVA. For abbreviations and symbols, see Tables S1 and S2.

Top-ranked metabolites correlating with the first component, statistically significant at CV-ANOVA (*n* = 35, abs(*p*(corr)[1]) ≥ 0.824, Figure 5a) were as follows: NAA (abs(*p*(corr)[1]) = 0.975), BHB, ScI, SAH, MTA, GSH, CyG, Ala, Phe, Tyr, Trp, Cre, GSx, PrCar, ValCar, Spmd, BuCar, Bet, MyI, Leu, HpX, Tau, Prp, ADP, Ins, Ads, Urd, Ura, Pro, FFA, For, ATP, Asp, Ade, and Acn (abs(*p*(corr)[1]) = 0.824). Most metabolites increased but NAA, BHB, ScI, Prp, ADP, FFA, For, and Acn decreased.

Top-ranked transcripts correlating with the first component, statistically significant at CV-ANOVA (*n* = 17, abs(*p*(corr)[1]) ≥ 0824, Figure 5b), were as follows: *HK*1 (abs(*p*(corr)[1]) = 0.975), *HK2*, *SHMT1*, *PFKFB3*, *GOT2*, *PTDSS1*, *IDH2*, *PCYT1A*, *ARG2*, *CDS1*, *SLC2A1*, *LDHA*, *ODC1*, *CTPS2*, *CHKA*, *TKT*, and *GPX1* (abs(*p*(corr)[1]) = 0.825). Most genes were downregulated but *PTDSS1* and *PCYT1A* were upregulated.

### 3.4. Joint Metabolite/Transcript Analysis Using ORA Identifies Most Important Pathways of Metabolic Reprogramming

While O2PLS identifies the most important genes and metabolites, ORA identifies the most important pathways from unsigned variations in metabolites and/or transcripts and the collection of metabolic maps of the KEGG database. ORA was performed using metabolites only, genes only, and joint metabolites/genes.

**Figure 6:**
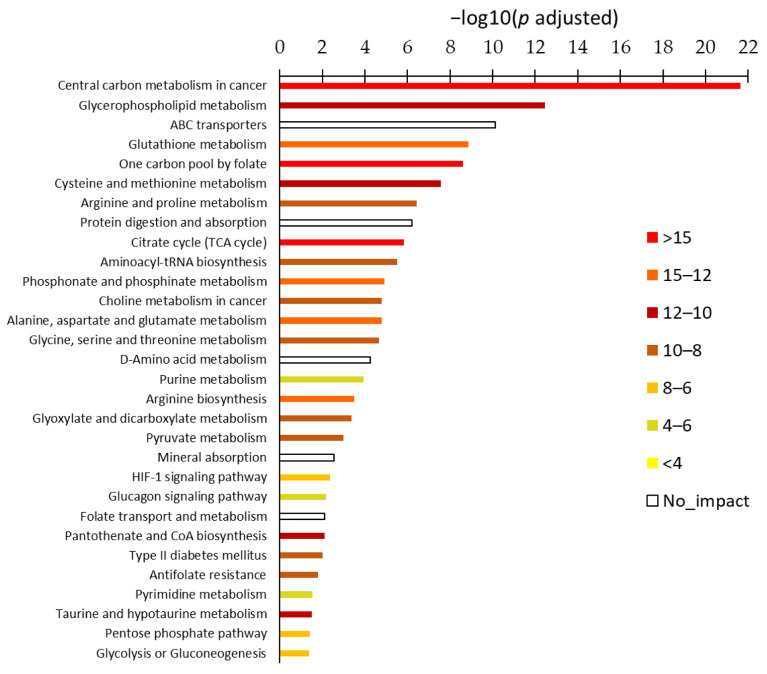
ORA of joint metabolites/genes showing statistically significant (−log10(*p*) > 1.3 at Holm adjustment) metabolism sets colored according to enrichment (right panel).

ORA driven by metabolites provided 6 statistically significant metabolism sets at Holm-adjusted geometrical test (Table S3) and ORA driven by genes, 12 statistically significant metabolism sets (Table S4). ORA of joint metabolites/genes provided 30 statistically significant metabolism sets (Figure 6, Table S5). The latter analysis was the most satisfactory since it accounted for the largest number of the most important metabolites and genes identified by O2PLS.

ORA reported the cancerous nature of the cell type through two pathways: ‘Central carbon metabolism in cancer’ and ’Choline metabolism in cancer’. Besides assignment of metabolites/genes to cancer pathology and after eliminating pathways with no impact, the first ranked biochemical pathways were ‘Glycerophospholipid metabolism’, ‘Glutathione metabolism, ‘One-carbon pool by folate’, ‘Cysteine and methionine metabolism’, ‘Arginine and proline metabolism’, and ‘Citrate cycle (TCA)’. In the middle of the list, we had pathways that obviously connected with the first ones including ‘Alanine, aspartate, and glutamate metabolism’, ‘Glycine, serine, and threonine metabolism’, ‘Purine metabolism’, ‘Arginine biosynthesis’, and pathways of carboxylic acids. Then, an unexpected but important result was the statistically significant ‘HIF-1 signaling pathway’. Actually, it is a classical way in molecular biology to identify the implication of a signaling pathway from the expression of one or a few canonical genes (including metabolism-related genes). The KEGG database indicates that metabolism-related target genes of the transcriptional factor HIF-1 are as follows: *HK1*, *HK2*, *PFKFB3*, *LDHA*, and *SLC2A1*. The expression of these five genes was significantly decreased, and these genes were collectively related to HIF-1 signaling downregulation. In addition, a metabolite also participated in the HIF-1 signaling pathway, namely ATP, which was increased. The pathway ranked quite late in the list of significant pathways, but this was due to the abundance of molecules of the pathway that did not belong to metabolism but were accounted for in the geometrical test (Figure 6).

ORA metabolism sets accounted for all genes identified as statistically significant with O2PLS. However, not all metabolites identified as significant with O2PLS were assigned to significant metabolism sets, including BHB, Acn, Prp, ScI, and MyI. Actually, these results were due to the very low relative abundance of probed vs. background molecules in the concerned metabolism sets. Therefore, a few other pathways, namely ‘Butanoate metabolism’ and ‘Inositol phosphate metabolism’, were retained retrospectively.

### 3.5. Combining Results of Joint Data Processing

In an attempt to make interpretation easier, we combined metabolism sets into supersets.

(1) A first metabolism superset was centered around bioenergetic metabolism and contained 7 pathways (‘Central carbon metabolism in cancer’, ‘Citrate cycle (TCA cycle)’, ‘Pyruvate metabolism’, ‘HIF-1 signaling pathway’, ’Pentose phosphate pathway’, ‘Glycolysis or Gluconeogenesis’, and ‘Butanoate metabolism’), 7 downregulated genes (*SLC2A1*, *HK1*, *HK2*, *PFKFB3*, *LDHA*, *IDH2*, and *TKT*), 8 increased metabolites (Ala, Asp, Leu, Phe, Pro, Tyr, and ATP) and 3 decreased metabolites (For, Acn, and BHB).
(2) A second metabolism superset was centered around glycerophospholipid metabolism and contained 3 pathways (‘Glycerophospholipid metabolism’, ‘Choline metabolism in cancer’, and ‘Inositol phosphate metabolism’), 2 upregulated genes (*PTDSS1* and *PCYT1A*), 2 downregulated genes (*CDS1* and *CHKA*), 1 increased metabolite (MyI), and 2 decreased metabolites (Sci and FFA).
(3) A third metabolism superset was centered around glutathione metabolism and contained 3 pathways (‘Glutathione metabolism’, ‘Cysteine and methionine metabolism’, and ‘Taurine and hypotaurine metabolism’), 5 downregulated genes (*GOT2*, *LDHA*, *IDH2*, *ODC1*, and *GPX1*), 9 increased metabolites (GSH, GSx, CyG, Spmd, Asp, MTA, SAH, Ala, and Tau) and 1 decreased metabolite (Ace).
(4) A fourth metabolism superset was centered around nucleic acid metabolism and contained 4 pathways (‘One-carbon pool by folate’, ‘Purine metabolism’, ‘Pantothenate and CoA biosynthesis’ and ‘Pyrimidine metabolism’), 2 downregulated genes (*SHMT1* and *CTPS2*), 10 increased metabolites (Asp, Ade, HpX, Ins, Ads, Ura, Urd, ATP, Bet, and SAH) and 1 decreased metabolite (ADP).
(5) A fifth metabolism superset was centered around Arg metabolism and contained 5 pathways (‘Arginine and proline metabolism’, ‘Alanine, aspartate, and glutamate metabolism’, ‘Glycine, serine, and threonine metabolism’, Arginine biosynthesis’, and ‘Glyoxylate and dicarboxylate metabolism’), 4 downregulated genes (*ARG2*, *ODC1*, *GOT2*, and *SHMT1*), 7 increased metabolites (Pro, Spmd, Cre, Bet, Trp, Asp, and Ala), and 2 decreased metabolites (NAA and For).

Then, in combination with KEGG database metabolism maps, a synthesis attempt was performed on enzyme regulations from metabolite and transcript fold variation.

The first metabolism superset contained evidence of bioenergetic metabolism changes including downregulation of glycolysis and HIF-1 signaling pathway. The increase in ATP (×2.41) and the consequences of HIF-1 inhibition on mitochondrial metabolism are in favor of activation of the oxidative TCA cycle and oxidative phosphorylation. This was accompanied by accumulation of amino acids testifying of increased synthesis from intermediates of the TCA cycle and/or inhibition of their catabolism. With these changes, we associated decreased levels of BHB and Acn, two ketone bodies.

The second metabolism superset demonstrated rewired glycerophospholipid metabolism. This BRCA1-induced metabolic alteration was not reported yet (Figure S1). There was evidence at the transcriptional level of increased PtS synthesis from base exchange with PtC through upregulation of *PTDSS1*, probably to support PtE synthesis. This reaction formed a loop with the second step of the CDP-choline pathway that was transcriptionally upregulated (*PCYT1A*). This allowed synthesis of PtC from PC and diacylgylcerol. *CHKA* was downregulated, probably explaining increased oxidation of choline in Bet. Another most important gene, *CDS1*, was downregulated, favoring the use of diacylglycerol for PtC synthesis. The important metabolite MyI was increased and reached high concentration in BRCA1-expressing cells.

The third reported superset contained evidence of activation of antioxidation processes with increased GSH, GSx, CyG, and downregulated *GPX1*.

The fourth superset contained elements for decreased de novo synthesis of nucleic acids and activation of the purine salvage pathway. A mechanism at the origin of reduced de novo synthesis of nucleic acid is inhibition of SHMT1 that reduces serine entry into the cytosolic one-carbon pool. Thus, the fate of serine was key in BRCA1-induced reprogramming since it was directed towards production of GSH and PtS under the dependence of the two most important genes *SHMT1* and *PTDSS1.*

The fifth metabolism superset contained evidence of rewired Arg metabolism including increased Cre and polyamine (Spmd) synthesis regulated by genes of polyamine synthesis (*ARG2*, *ODC1*), all closely correlated. Based on increased products of Arg metabolism (Cre, Spmd, and MTA), it may be concluded that there was increased Arg metabolism. This BRCA1-induced metabolic alteration was not reported yet (Figure S2).

## 4. Discussion

To unravel the extent of metabolic reprogramming of BRCA1-expressing breast cancer cells, we used several untargeted metabolomics platforms and transcriptional expression of metabolism-related genes. We analyzed data by two joint metabolite/transcript data processing, namely O2PLS using SIMCA 14 and pathway analysis using MetaboAnalyst. The combined results allowed us to highlight major traits of metabolism reprogramming induced by stable BRCA1 expression in breast cancer cells. Some of them were not yet reported. Briefly, the strength of performing joint (O2PLS) vs. sequential data processing is that produced correlation coefficients are more reliable due to the removal of structured noise and influence of other variables. The strength of ORA as a joint vs. sequential data processing lies in improved statistics and ranking.

In this study, we used a single breast cancer cell line, namely SUM1315MO2, classified as TNBC. Since cancer cells are known for exhibiting cell-type-dependent behavior, it may be questioned whether reported results on BRCA1-induced metabolic reprogramming may be generalized to other cancer cell types. Actually, previous studies on BRCA1-induced metabolic alterations have reported in numerous cell lines (breast, ovarian, and others) metabolic features that we also found in this study (see below). We thus consider that the used breast cancer cell line was representative of general BRCA1-induced metabolic reprogramming.

The extent of observed transcriptional changes may be related to the fact that the BRCA1 protein is expected to interact with proteins recognizing gene promoters, thus behaving as a coregulator of transcription [1]. This was recently demonstrated with a gene of metabolism, *GOT2*, regulated by a complex formed by BRCA1 with a zinc finger protein [11]. Besides transcriptional coregulation activity, the BRCA1 protein directly interacts with ACC1, thus inhibiting the enzyme and decreasing de novo FA synthesis [2]. Due to a particular mechanism, the consequences of this action may precede BRCA1-induced transcriptional coregulation. Specifically, ACC1 inhibition will alter intermediary metabolites including acetyl-CoA (ACoA), Cit, and alphaketoglutarate (AKG) [19,20]. Since ACC1 produces malonyl-CoA from two molecules of ACoA, its inhibition will cause an increase in ACoA cytosolic levels. This yields to histone acetylation that makes genes more accessible to transcriptional regulation [19]. Thus, ACC1 inhibition may predispose BRCA1-expressing breast cancer cells for transcriptionally based reprogramming. Then, upstream of ACoA, other intermediary metabolites Cit and AKG should accumulate [20]. Consistently, we found, in BRCA1-expressing cancer cells, an increase in Cit at univariate statistics (×1.83). Then the increase in cytosolic AKG upstream Cit should impact AKG-dependent dioxygenases, including the most investigated ones such as HIF-1 prolylhydroxylases and Jumonji C-domain-containing histone demethylases. Thus, it may be expected that there is a decrease in HIF-1 protein levels as well as a lowering of global histone/DNA methylation in BRCA1-expressing cancer cells. These remarks are in line with the metabolic alterations discussed below.

The first major trait of metabolic reprogramming of BRCA1-expressing tumor cells in this study was bioenergetic metabolism alteration. Downregulation of glycolysis and activation of oxidative phosphorylation are reported by several studies [6,7,8]. Pathway analysis provided a cause for bioenergetics changes: HIF-1 signaling pathway decreased activity. HIF-1 is known for mediating responses to hypoxia. The regulation of HIF-1 principally depends on protein stabilization by inactivation of prolylhydroxylases that belong to the family of AKG-dependent dioxygenases. In contrast, once activated, prolylhydroxylases engage the HIF-1 protein in a degradation process. HIF-1 alpha was found to be more frequently overexpressed in *BRCA1*-mutated breast cancers [9]. Mechanistically, BRCA1 was shown to regulate HIF-1 stability [10]. Besides that, ACC1 inhibition, independently of BRCA1 expression, causes a rise in intermediary metabolites ACoA and AKG, increases global histone acetylation [19,20,21], and decreases the activity of HIF-1 [20]. Therefore, it may be questioned what the role of AKG has in the decreased activity of HIF-1 in BRCA1-expressing cells.

Also, the TCA cycle was reprogrammed as an oxidative pathway. A gene whose protein belonged to the TCA, *IDH2*, was identified as most important and downregulated. IDH2 is a bidirectional enzyme and catalyzes the interconversion of isocitrate and AKG using NADPH as a cofactor. In normal cells, IDH2 mostly catalyzes the oxidative decarboxylation of isocitrate into AKG. However, in tumor cells, reductive carboxylation of AKG may occur through IDH2 [22] or its cytosolic isoform IDH1 [23]. This pathway is the primary route through which glutamine, Glu, and AKG are converted to Cit and then to FA. A recent study using labeled glutamine experiments showed that suppression of *IDH2* in breast cancer cells decreased reductive carboxylation of AKG in the TCA cycle and increased AKG levels [24]. Thus, *IDH2* downregulation contributes to activation of oxidative TCA cycle. We also found, as a mitochondrial important gene, downregulation of *GOT2*. GOT2 intervenes in the malate–aspartate shuttle, which enables the transfer of reduced equivalents of NADH from the cytosol to the mitochondria. GOT2 produces AKG and Asp from Glu and oxaloacetic acid. *GOT2* downregulation should direct Glu towards other pathways including GSH metabolism. *GOT2* downregulation was in agreement with the recent report that BRCA1 formed with a zinc finger protein a complex able to repress the gene [11].

It may be assumed the decrease in FA beta-oxidation from the decrease in ketone bodies (BHB, Acn) and downregulated *CPT1A* at univariate statistics (×0.89). CPT1A is a cytosolic-mitochondrial transporter and the rate-limiting enzyme in mitochondrial FA oxidation by controlling the entry of cytosolic-produced free FA. Moreover, BHB functions as a histone deacetylase inhibitor, increases histone acetylation, and is growth-inhibitory [25]. Short chain acylcarnitines (PrCar, ButCar, and ValCar) were increased in SUM1315-BRCA1 cells. The origin of these metabolites is unclear but indicates intracellular trafficking of incompletely oxidized carbon chains [26].

Glycerophospholipid metabolism ranked at the top of altered metabolic pathways and was not reported yet as a consequence of BRCA1 expression (Figure S1). Among the most important genes related to glycerophospholipid metabolism were as follows: *PTDSS1*, *PCYT1A*, *CDS1*, and *CHKA*. From upregulation of *PTDSS1* (×1.78) (and downregulation of *PTDSS2* at univariate statistics), there was evidence of increased PtS synthesis from base exchange with PtC, whereas base exchange with PtE was spared. Base exchange is the regular pathway for PtS synthesis, which, in turn, is the precursor for PtE synthesis by decarboxylation in mitochondria. Actually, PtE is an abundant phospholipid in mitochondrial membranes, and such metabolic regulation is a requirement for mitochondrial dynamics. PtC is resynthesized from the CDP-choline pathway that is upregulated (*PCYT1A*, ×1.99) and closely correlated with *PTDSS1* expression. CDS1 was downregulated (×0.62), which favored synthesis of PtC from diacylglycerol and PC through the CDP-choline pathway, consistent with the fact that PtI and MyI levels were elevated. Choline released by base exchange should not be fully recycled as PC to synthesize PtC since *CHKA* was downregulated (×0.78). The origin of CHKA regulation is multiple and implicates several oncogenic pathways as shown by the KEGG database. However, choline was partly oxidized as Bet a most important metabolite (×1.96).

An unexpected finding was the accumulation of free MyI (×2.83) that inversely correlated with ScI (×0.34). MyI is known for its implication as an osmolyte in hypertonic environments and as a source of phosphoinositides in signal transduction. In our cancer cell model, the origin of MyI cannot be specified and may relate to uptake from the medium, production from glucose-6-P, or upregulation of inositol monophosphatases 1/2. Recently, MyI was shown to have a signaling role as a free molecule. The role of MyI could be that it binds to and restricts activation of AMP-activated protein kinase (AMPK) to maintain mitochondrial dynamics [27]. The regulation of mitochondrial fission and fusion is key in SUM1315-BRCA1 cells that rely on oxidative phosphorylation. Another most important gene of the phospholipid metabolism was downregulated, *CDS1*. In BRCA1-expressing cells, *CDS1* downregulation as well as the increased levels of PtI (×1.41) and of MyI favor PtC or PtE synthesis from phosphatidic acid.

We found evidence of upregulation of antioxidation processes (downregulation of *GPX1* and increased levels of GSH, GSx, and CyG at CV-ANOVA, as well as upregulation of *GCLC* (×1.82) at *t*-test). Most of these molecules are implicated in GSH synthesis. GPX1 utilizes GSH as a substrate to catalyze peroxides and generates GSSG. NADPH, a necessary cofactor for GSH maintenance, was probably supplied by IDH1, which was upregulated (×1.63) at univariate statistics. The negative expression of *GOT2* covaried with increased amount of GSH and GyG suggesting that downregulation of *GOT2* played a role in increased GSH synthesis. Upregulation of antioxidant systems in BRCA1-expressing cells is in line with the literature. BRCA1 induced the expression of glutathione-S-transferase and other antioxidant genes and GSH synthesis in cancer cell lines [3,6]. It also induced expression of Nrf2-regulated antioxidant genes [5].

We found elements in favor of decreased de novo synthesis of nucleic acids and activation of the purine salvage pathway. Most important genes related to these pathways were *SHMT1* and *CTPS2*, which were downregulated. Purine nucleotides are synthesized through two pathways: salvage and de novo. The salvage pathway recycles nucleobases to produce mononucleotides. We found increased levels of Ins, Ade, and Ads (×3.6), as well as HpX (×7.0), whereas GMP was decreased at univariate statistics (×0.42), the latter probably in relation with downregulation of *IMPDH2* (×0.43). These data indicate downregulation of de novo purine nucleotide synthesis, at the benefit of the salvage pathway. In agreement with our findings, a metabolomics study identified Ade as a biomarker of the *BRCA1* genotype that was significantly increased in wild-type *BRCA1*-expressing breast cancer cells [15]. Pyrimidines can also be synthesized from de novo or salvage pathways. De novo pyrimidine nucleotide synthesis relies on TYMS that was downregulated at univariate statistics (×0.89), whereas the salvage pathway uses thymidine kinase 1 instead. We had an accumulation of Ura (×8.0) and Urd (×25) that may be explained by *CTPS2* downregulation in SUM1315-BRCA1 cells. These data support downregulation of de novo pyrimidine nucleotide synthesis possibly, also at the benefit of the salvage pathway.

A most important gene of the one-carbon metabolism, *SHMT1*, was downregulated (×0.68). SHMT1 catalyzes in the cytosol the conversion of serine and tetrahydrofolate (THF) to Gly and methylene-THF. *SHMT1* knockdown was shown to cause an accumulation of Ura during DNA replication and reduced dTMP synthesis [28]. *SHMT1* downregulation should make available one-carbon units or serine for other biosynthesis. BRCA1-expressing cells exhibit two pathways with increased serine consumption, namely PtS synthesis and GSH synthesis. Regarding other important metabolites of the ‘One-carbon metabolism by folates’ map, we had increased Bet and SAH. Bet is an oxidized form of choline and links the phospholipid metabolism to the methionine (Met) metabolism. Bet was increased due to downregulation of *CHKA* and *BHMT* (the latter at univariate statistics). SAH is a product common to all types of transmethylations and was increased (×3.04). In addition, there was evidence of increase in some transmethylations like GAMT (but not at the transcriptional level) causing an increase in Cre (×2.30) and GNMT at the transcriptional level (×4.48). Alternatively, BRCA1 expression was shown to negatively correlate with another methyltransferase, NNMT [8].

The fifth metabolic superset showed alteration in Asp, Arg, Pro, and Glu metabolism. Despite increase in cytosolic ACoA induced by ACC1 inhibition, NAA, an acetyl group acceptor, was decreased (×0.36). In this study, NAA was identified as a most important metabolite. A recent report in brown adipocytes showed that NAA was an important regulator of the ACoA pool and that NAA levels inversely correlated with transcriptional activity [29]. Thus, in BRCA1-expressing cells, decreased levels of NAA indicated that sources of cytosolic ACoA were downregulated including histone deacetylases and ATP-citrate lyase (*ACLY*, ×0.67, in this study).

Regarding the ‘Arginine and proline metabolism’ pathway (Figure S2), entries in the pathway were increased including Arg (×1.49), Pro (×2.48), and Glu (×1.98). Arg metabolism was increased as attested by increased downstream metabolites including Cre (×2.30) and polyamine (Spmd (×2.27) and Spm (×1.29)). To these metabolites, we can add MTA (×2.30), a product of polyamine synthesis and a most important metabolite in this study, appearing in the ‘Cysteine and methionine metabolism’ metabolic map. MTA is recycled into Met and Ade, and it contributes to Met and purine salvage pathways. Both *ARG2* and *ODC1* transcriptionally, and MTA allosterically, regulate polyamine synthesis. A rewiring of Glu metabolism may also contribute to polyamine synthesis since glutamine was kept away from FA production in BRCA1-expressing cancer cells.

## 5. Conclusions

Major traits of metabolic reprogramming were obtained by joint metabolomics/transcriptomics in BRCA1-expressing breast cancer cells. The breadth of BRCA1-induced metabolic reprogramming included decrease in intracellular FA levels, downregulation of glycolysis, decreased activity of the HIF-1 signaling pathway, upregulation of PtS synthesis from base exchange with PtC, upregulation of antioxidation processes, decreased de novo purine nucleotide synthesis, and increased Arg metabolism. Among these pathways, rewired glycerophospholipid and Arg metabolism have not been reported yet. In addition, it is shown that the fate of serine is key in BRCA1-induced reprogramming and directed towards GSH and PtS synthesis. The study may help to identify metabolites that are candidate biomarkers of the *BRCA1* genotype, understand global oncosuppressive effects of *BRCA1*, and identify metabolic vulnerabilities that can be exploited in targeted therapies.

## Data Availability

The original contributions presented in this study are included in the article and Supplementary Material.

## Supplementary Materials

The following supporting information can be downloaded at: https://www.mdpi.com/article/10.3390/metabo15080534/s1. Figure S1: The ‘Glycerophospholipid metabolism’ map of the KEGG database with the report of metabolite and transcript changes in BRCA1-expressing breast cancer cells; Figure S2: The ‘Arginine and proline metabolism’ map of the KEGG database with the report of metabolite and transcript changes in BRCA1-expressing breast cancer cells; Table S1: Quantified metabolites from multiple platforms; Table S2: Quantified transcripts; Table S3: ORA driven by metabolites; Table S4: ORA driven by genes; Table S5: ORA driven by metabolites/genes.

## Abbreviations

The following abbreviations are used in this manuscript:

*BRCA1*/BRCA1	Breast cancer susceptibility gene 1 (gene/protein)
FA	Fatty acid
ACC1	Acetyl-CoA carboxylase 1
ROS	Reactive oxygen species
HIF-1	Hypoxia-inducible-factor 1
GOT2	Glutamic-oxaloacetic transaminase 2
NNMT	Nicotinamide N-methyltransferase
Ade	Adenine
PtS	Phosphatidylserine
PtC	Phosphatidylcholine
GSH/GSSG	Reduced/oxidized glutathione
Arg	Arginine
TNBC	Triple-negative breast cancer
Cre	Creatine
OPLS	Orthogonal partial least squares
CV-ANOVA	Cross-validated ANOVA
O2PLS	Two-way orthogonal partial least squares
ORA	Overrepresentation analysis
GSx	Total glutathione
tCr	Total creatine
MyI	Myoinositol
Tau	Taurine
Glu	Glutamic acid
BHB	3-Hydroxybutyric acid
NAA	N-acetyl-L-aspartic acid
PC	Phosphorylcholine
ScI	Scylloinositol
TCA	Tricarboxylic acid cycle
ACoA	Acetyl-CoA
AKG	Alphaketoglutarate
NADH	Reduced nicotinamide adenine dinucleotide
NADPH	Reduced nicotinamide adenine dinucleotide phosphate
AMPK	AMP-activated protein kinase
