# Supplementary material for: Joint Metabolomics and Transcriptomics Reveal Rewired Glycerophospholipid and Arginine Metabolism as Components of BRCA1-Induced Metabolic Reprogramming in Breast Cancer Cells
Source: Metabolites. 2025 Aug 7;15(8):534. doi: 10.3390/metabo15080534 (PMC12388146; doi:10.3390/metabo15080534)
Supplement: Supplementary file 1 [file metabolites-15-00534-s001.zip › metabolites-3778644 Supplementary.pdf]

metabolites

MDPI

Joint metabolomics and transcriptomics reveal rewired glycerophospholipid and arginine metabolism as components of BRCA1-induced metabolic reprogramming in breast cancer cells

Thomas Lucaora and Daniel Morvan

Supplementary Materials: Figure S1: The 'Glycerophospholipid metabolism' map of the KEGG database with the report of metabolite and transcript changes in BRCA1-expressing breast cancer cells; Figure S2: The 'Arginine and proline metabolism' map of the KEGG database with the report of metabolite and transcript changes in BRCA1-expressing breast cancer cells; Table S1: Quantified metabolites from multiple platforms; Table S2: Quantified transcripts; Table S3: ORA driven by metabolites; Table S4: ORA driven by genes; Table S5: ORA driven by metabolites/genes.

.

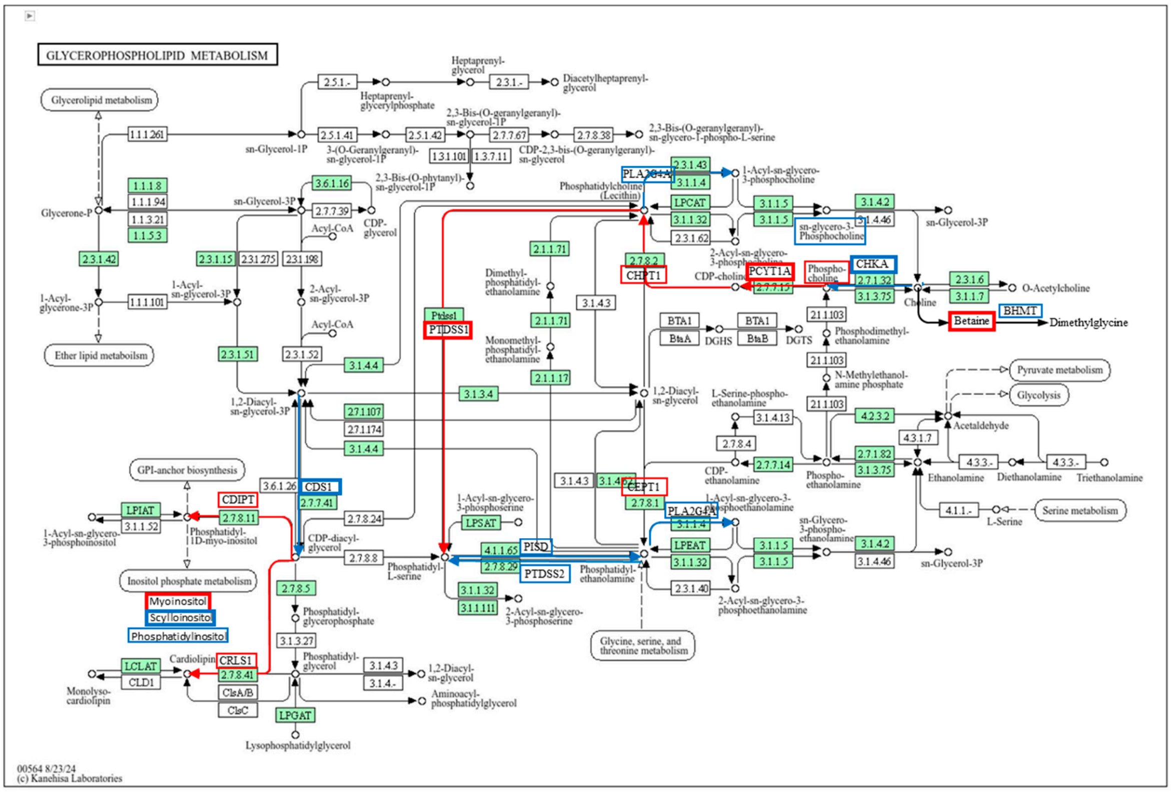

Figure S1: The ‘Glycerophospholipid metabolism’ map of the KEGG database with the report of metabolite and transcript changes in BRCA1-expressing breast cancer cells. Box, Metabolite or transcript with statistically significant variation. Thick/thin, Significant at CV-ANOVA/t-test. Red, Increased. Blue, Decreased.

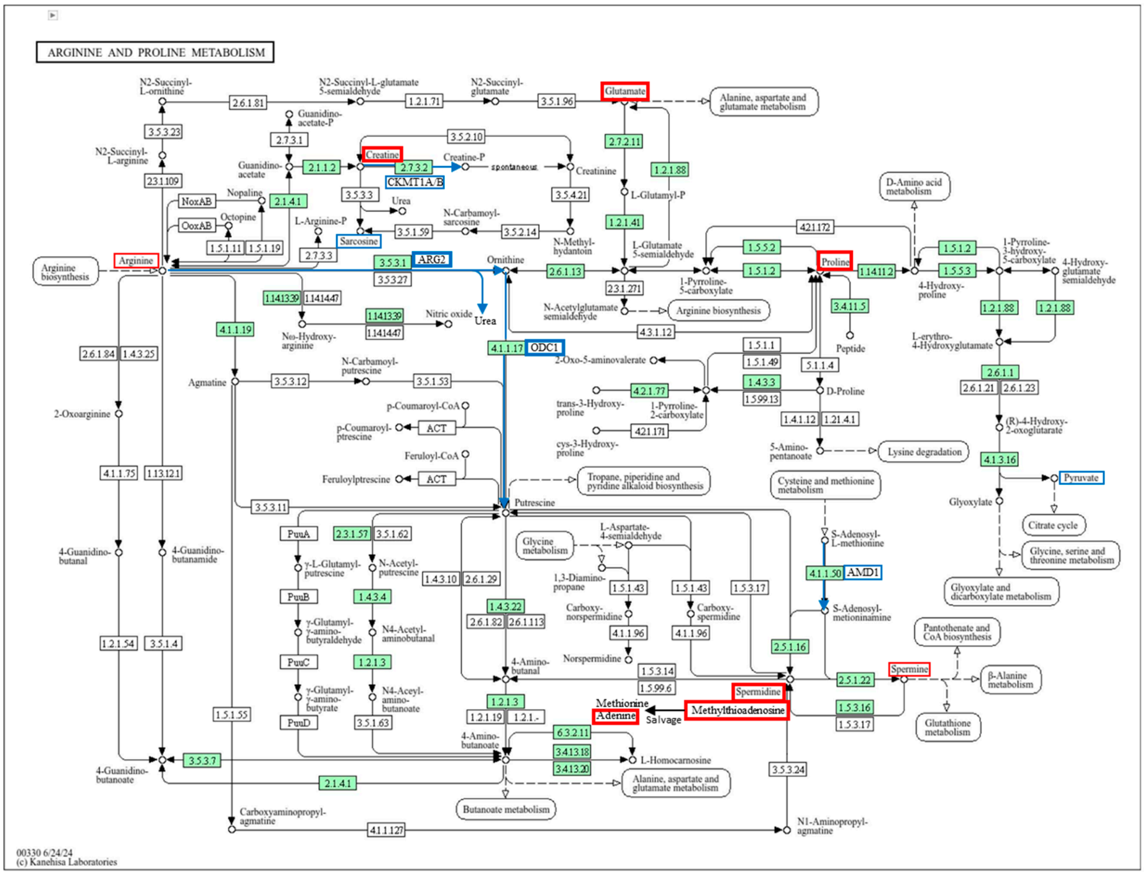

Figure S2. The 'Arginine and proline metabolism' map of the KEGG database with the report of metabolite and transcript in BRCA1-expressing breast cancer cells. Box, Metabolite or transcript with statistically significant variation. Thick/thin border, Significant at CV-ANOVA/t-test. Red, Increased. Blue, Decreased.

Table S1. Quantified metabolites from multiple platforms. Abbreviation, full name and fold variation (SUM1315-BRCA1, *n*=10, vs. SUM1315-CT, *n*=8)

**Metabolite abbreviation**	**Full name**		**Fold variation**	**SD**	***p* value (Student's t-test)**
NMR spectroscopy		Chemical shift (PPM)			
Ace	Acetic acid	1.96	0.52	0.1	3.50E-05
Acn	Acetone	2.23	0.51	0.19	1.97E-04
Ads(MR)	Adenosine	8.35,8.23,6.10	2.69	1.89	2.45E-02
Asp	L-Aspartic acid	2.65,2.82	5.75	2.25	2.26E-05
ATP(MR)	Adenosine triphosphate	8.54,8.26,6.15	1.72	0.47	6.93E-04
BHB	3-Hydroxybutyric acid	1.18,2.35,4.15	0.17	0.05	3.86E-12
For	Formic acid	8.46	0.43	0.17	4.88E-06
Glu(MR)	Glutamic acid	2.08,2.35,2.40,3.75	1.26	0.28	3.01E-02
Gly	Glycine	3.56	2.26	0.6	1.19E-04
GPC	Glycerophosphocholine	3.68,3.23	0.58	0.06	2.88E-04
GSx	Total glutathione	2.16,2.55,2.96,3.77	1.88	0.15	1.72E-08
Lac	Lactic acid	1.33,4.12	0.82	0.24	2.40E-01
MyI	Myo-Inositol	3.25,3.53,3.62,4.05	2.83	0.73	4.10E-06
NAA	N-Acetyl-L-aspartic acid	2.07,2.49,2.68	0.36	0.06	3.32E-11
PC	Phosphorylcholine	4.18,3.62,3.22	1.61	0.52	7.39E-03
Prp	Propionic acid	1.06,2.17	0.64	0.09	1.29E-06
Pyr	Pyruvic acid	2.4	0.45	0.1	9.54E-06
Sarc	Sarcosine	2.73,3.60	0.6	0.17	4.44E-03
ScI	Scyllo-Inositol	3.36	0.34	0.06	1.77E-09
Succ	Succinic acid	2.39	0.32	0.2	5.13E-04
Tau	Taurine	3.27,3.43	2.03	0.33	3.29E-06
tCr	Total creatine	3.035,3.93	1.6	0.24	6.67E-05
LCMS					
AcCar	L-Acetylcarnitine		1.3	0.26	1.24E-02
Ade	Adenine		2.57	0.83	7.67E-05
Ads	Adenosine		3.57	1.17	1.40E-05
ADP	Adenosine 3',5'-diphosphate		0.74	0.07	2.86E-07
Ala	L-Alanine		2.34	0.28	1.31E-08
AMP	Adenosine monophosphate		0.74	0.22	7.86E-02
Arg	L-Arginine		1.49	0.39	6.50E-03
ATP	Adenosine triphosphate		2.41	0.57	4.30E-06
Bet	Betaine		1.96	0.27	3.79E-07
BuCar	Butyryl-L-carnitine		6.76	2.15	1.23E-06

Cit	Citric acid	1.83	0.53	2.67E-03
Cre	Creatine	2.3	0.33	8.01E-08
Cyd	Cytidine	2.51	1.82	3.60E-02
CyGly	Cysteinylglycine	2.23	0.25	3.56E-09
Cyt	Cytosine	6.16	4.85	9.28E-03
DHO	4,5-Dihydroorotic acid	0.63	0.12	1.08E-05
Glu	Glutamic acid	1.98	0.66	8.95E-04
GMP	Guanosine monophosphate	0.42	0.16	2.44E-05
Gns	Guanosine	2.65	0.99	5.99E-04
GSH	Glutathione	1.9	0.11	1.27E-10
GSSG	Oxidized glutathione	1.11	0.25	3.07E-01
Gua	Guanine	2.58	0.92	4.17E-04
HpX	Hypoxanthine	7.03	2.36	3.43E-06
IMP	Inosinic acid	0.32	0.17	1.64E-02
Inos	Inosine	5.92	2	4.79E-06
Leu	Leucine	1.84	0.24	1.48E-06
Met	Methionine	1.22	0.24	7.05E-02
MTA	5'-Methylthioadenosine	2.3	0.27	1.20E-09
OP	Pyroglutamic acid	1.15	0.35	3.41E-01
Phe	Phenylalanine	2.02	0.26	8.63E-08
PrCar	Propionylcarnitine	2.78	0.65	1.58E-06
Pro	Proline	2.48	0.48	1.27E-06
SAH	S-Adenosylhomocysteine	3.04	0.46	2.49E-09
Spm	Spermine	1.29	0.16	6.39E-04
Spmd	Spermidine	2.27	0.45	9.52E-07
Trp	L-Tryptophan	1.86	0.19	1.96E-08
Tyr	L-Tyrosine	1.82	0.22	5.62E-08
Urd	Uridine	25	10.74	3.21E-05
Ura	Uracil	8	3.23	1.59E-05
Val	L-Valine	1.39	0.15	1.59E-04
ValCar	Valerylcarnitine	4.86	1.28	3.05E-07
HPTLC				
FFA	Free fatty acid(s)	0.38	0.18	8.82E-06
PtC	Phosphatidylcholine(s)	0.9	0.26	3.93E-01
PtE	Phosphatidylethanolamine(s)	1.24	0.29	8.50E-02
PtI	Phosphatidylinositol(s)	1.41	0.34	1.18E-02
PtS	Phosphatidylserine(s)	1.15	0.23	1.80E-01
SGM	Sphingomyelin(s)	0.78	0.15	2.61E-02

Table S2. Quantified transcripts. Gene symbol, full name and transcript fold variation (SUM1315-BRCA1, *n*=10, vs. SUM1315-CT, *n*=8)

**Gene symbol**	**Full name**	**Protein short name**	**Fold variation**	**SD**	***p* value (Student's t-test)**

ACACA	Acetyl-CoA carboxylase alpha	ACC1	0.79	0.16	4.39E-02
ACLY	ATP citrate lyase	ACLY	0.67	0.1	3.83E-04
ACO1	Aconitase 1	ACO1	0.8	0.1	3.99E-03
ACO2	Aconitase 2	ACO2	0.99	0.18	9.68E-01
AGXT	Alanine-glyoxylate and serine-pyruvate transaminase	AGXT	1.29	0.48	2.63E-01
AMD1	Adenosylmethionine decarboxylase 1	AMD1	0.89	0.09	4.90E-02
ARG2	Arginase 2	ARG2	0.56	0.07	3.20E-07
ASNS	Asparagine synthetase (glutamine-hydrolyzing)	ASNS	0.91	0.19	2.55E-01
BHMT	Betaine-homocysteine S-methyltransferase	BHMT	0.45	0.12	3.95E-02
CAD	Carbamoyl-phosphate synthetase 2, aspartate transcarbamylase, and dihydroorotase	CAD	1.05	0.18	5.06E-01
CBS	Cystathionine beta-synthase	CBS	1.02	0.15	7.97E-01
CDIPT	CDP-diacylglycerol-inositol 3-phosphatidyltransferase	CDIPT	1.23	0.08	1.61E-05
CDS1	CDP-diacylglycerol synthase 1	CDS1	0.62	0.07	1.74E-08
CEPT1	Choline/ethanolamine phosphotransferase 1	CEPT1	1.08	0.06	4.28E-02
CHKA	Choline kinase alpha	CHKA	0.78	0.1	2.74E-04
CHPT1	Choline phosphotransferase 1	CHPT1	1.6	0.37	5.13E-04
CKB	Creatine kinase B	CKB	1.18	0.34	2.33E-01
CKMT1A/B	Creatine kinase, mitochondrial 1A/B	CKMT1A/B	0.4	0.06	3.63E-03
CPT1A	Carnitine palmitoyltransferase 1A	CPT1A	0.89	0.11	4.34E-02
CRLS1	Cardiolipin synthase 1	CRLS1	1.25	0.13	3.72E-04
CS	Citrate synthase	CS	0.88	0.18	1.48E-01
CTPS2	CTP synthase 2	CTPS2	0.71	0.09	1.81E-05
CYP4F3	Cytochrome P450 family 4 subfamily F member 3	CYP4F3	1.73	1.07	1.62E-01
DGKA	Diacylglycerol kinase alpha	DGKA	1.1	0.08	1.97E-01
DGKG	Diacylglycerol kinase gamma	DGKG	3.28	2.95	6.87E-02
ETNK1	Ethanolamine kinase 1	ETNK1	0.91	0.1	6.93E-02
FADS1	Fatty acid desaturase 1	FADS1	0.92	0.18	4.26E-01
FASN	Fatty acid synthase	FASN	1.45	0.24	1.94E-04
FH	Fumarate hydratase	FH	0.74	0.18	2.35E-03
G6PD	Glucose-6-phosphate dehydrogenase	G6PD	0.86	0.08	1.83E-03
GAMT	Guanidinoacetate N-methyltransferase	GAMT	1.05	0.14	3.89E-01
GCLC	Glutamate-cysteine ligase catalytic subunit	GCLC	1.82	0.3	6.49E-06
GLS	Glutaminase	GLS	0.87	0.1	1.39E-01
GNMT	Glycine N-methyltransferase	GNMT	4.48	2.15	4.39E-04
GOT1	Glutamic-oxaloacetic transaminase 1	GOT1	1.03	0.14	6.60E-01
GOT2	Glutamic-oxaloacetic transaminase 2	GOT2	0.45	0.02	8.95E-10

GPAM	Glycerol-3-phosphate acyltransferase, Mitochondrial	GPAM	1.08	0.19	2.78E-01
GPT	Glutamic-pyruvic transaminase	GPT	1.47	0.86	1.74E-01
GPT2	Glutamic-pyruvic transaminase 2	GPT2	0.86	0.14	5.78E-02
GPX1	Glutathione peroxidase 1	GPX1	0.81	0.06	3.69E-05
GSR	Glutathione-disulfide reductase	GSR	1.1	0.09	1.93E-02
HK1	Hexokinase 1	HK1	0.46	0.04	1.04E-10
HK2	Hexokinase 2	HK2	0.43	0.06	9.54E-09
HMGCR	3-Hydroxy-3-methylglutaryl-CoA reductase	HMGCR	0.92	0.11	1.54E-01
IDH1	Isocitrate dehydrogenase (NADP(+)) 1	IDH1	1.63	0.21	2.83E-06
IDH2	Isocitrate dehydrogenase (NADP(+)) 2	IDH2	0.53	0.06	2.26E-07
IMPDH2	Inosine monophosphate dehydrogenase 2	IMPDH2	0.43	0.17	4.28E-03
LDHA	Lactate dehydrogenase A	LDHA	0.71	0.08	5.91E-05
MAT2B	Methionine adenosyltransferase 2B	MAT2B	0.86	0.07	3.97E-03
MDH1	Malate dehydrogenase 1	MDH1	0.92	0.07	1.09E-01
MDH2	Malate dehydrogenase 2	MDH2	0.97	0.06	3.57E-01
NOS2	Nitric oxide synthase 2	NOS2	2.4	2.67	2.03E-01
NOX1	NADPH oxidase 1	NOX1	1.45	1.04	2.79E-01
ODC1	Ornithine decarboxylase 1	ODC1	0.75	0.06	1.19E-05
OTC	Ornithine carbamoyltransferase	OTC	0.64	0.23	1.32E-02
PCYT1A	Phosphate cytidylyltransferase 1, choline, alpha	PCYT1A	1.99	0.15	8.09E-11
PCYT1B	Phosphate cytidylyltransferase 1, choline, beta	PCYT1B	0.33	0.21	1.49E-02
PCYT2	Phosphate cytidylyltransferase 2, ethanolamine	PCYT2	1.19	0.12	4.71E-03
PDHA1	Pyruvate dehydrogenase E1 subunit alpha 1	PDHA1	0.83	0.19	1.87E-01
PEMT	Phosphatidylethanolamine N-methyltransferase	PEMT	1.11	0.18	1.50E-01
PFKFB3	6-Phosphofructo-2-kinase/fructose-2,6-biphosphatase 3	PFKFB3	0.54	0.09	9.30E-07
PFKL	Phosphofructokinase, liver type	PFKL	0.88	0.09	2.04E-02
PGAM4	Phosphoglycerate mutase family member 4	PGAM4	0.88	0.35	5.26E-01
PISD	Phosphatidylserine decarboxylase	PISD	0.8	0.17	1.04E-02
PKM	Pyruvate kinase M1/2	PKM	0.83	0.12	8.84E-03
PLA2G4A	Phospholipase A2 group IVA	PLA2G4A	0.85	0.08	3.96E-02
PLA2G6	Phospholipase A2 group VI	PLA2G6	1.07	0.25	5.51E-01
PLCG1	Phospholipase C gamma 1	PLCG1	0.84	0.14	2.48E-02
PLD1	Phospholipase D1	PLD1	1.12	0.14	6.76E-02
PSAT1	Phosphoserine aminotransferase 1	PSAT1	1.01	0.21	9.35E-01
PTDSS1	Phosphatidylserine synthase 1	PTDSS1	1.78	0.11	4.66E-11

PTDSS2	Phosphatidylserine synthase 2	PTDSS2	0.77	0.12	4.80E-03
PTGS2	Prostaglandin-endoperoxide synthase 2	PTGS2	1.91	0.57	8.61E-04
RPE	Ribulose-5-phosphate-3-epimerase	RPE	1.38	0.16	1.80E-05
RPIA	Ribose 5-phosphate isomerase A	RPIA	1.1	0.11	5.71E-02
RRM1	Ribonucleotide reductase catalytic subunit M1	RRM1	0.85	0.08	2.33E-03
SDHC	Succinate dehydrogenase complex subunit C	SDHC	1.26	0.17	1.67E-03
SGMS1	Sphingomyelin synthase 1	SGMS1	1.02	0.1	6.47E-01
SGMS2	Sphingomyelin synthase 2	SGMS2	0.89	0.05	7.00E-04
SGPL1	Sphingosine-1-phosphate lyase 1	SGPL1	1.04	0.13	6.37E-01
SHMT1	Serine hydroxymethyltransferase 1	SHMT1	0.68	0.04	7.36E-11
SHMT2	Serine hydroxymethyltransferase 2	SHMT2	1.05	0.11	3.42E-01
SLC2A1	Solute carrier family 2 member 1	SLC2A1,	0.42	0.08	3.65E-06
SPHK1	Sphingosine kinase 1	SPHK1	1.09	0.21	2.58E-01
SPTLC1	Serine palmitoyltransferase long chain base subunit 1	SPTLC1	0.92	0.11	2.21E-01
SQLE	Squalene epoxidase	SQLE	1.21	0.1	3.56E-04
TALDO1	Transaldolase 1	TALDO1	1.04	0.11	4.73E-01
TKT	Transketolase	TKT	0.81	0.08	2.39E-05
TYMS	Thymidylate synthetase	TYMS	0.87	0.08	1.92E-03
UGDH	UDP-glucose 6-dehydrogenase	UGDH	1.03	0.16	6.58E-01

Table S3. ORA driven by metabolites

Metabolite set	Total	Expected	Hits	Raw *p*	Holm adjust	FDR	Impact
Glycerophospholipid metabolism	87	3.7271	20	1.6949E-10	1.3729E-08	1.3729E-08	0.62791
Glutathione metabolism	57	2.4419	14	4.9832E-08	3.9866E-06	2.0182E-06	0.82143
Phosphonate and phosphinate metabolism	10	0.4284	5	0.000027946	0.0022078	0.00056362	0.88889
One carbon pool by folate	72	3.0845	12	0.000036661	0.0028596	0.00056362	0.80282
Citrate cycle (TCA cycle)	42	1.7993	9	0.000047438	0.0036527	0.00056362	1.0732
Aminoacyl-tRNA biosynthesis	74	3.1702	12	0.000048708	0.0037018	0.00056362	0.16438
Arginine and proline metabolism	74	3.1702	12	0.000048708	0.0037018	0.00056362	0.41096
Arginine biosynthesis	28	1.1995	7	0.00012253	0.0090669	0.0012406	0.74074

Cysteine and methionine metabolism	72	3.0845	11	0.00017937	0.013094	0.0016143	0.5493

Table S4. ORA driven by genes

Gene set	Total	Expected	Hits	Raw *p*	Holm adjust	FDR	Impact
Glycerophospholipid metabolism	103	0.96511	17	1.6656E-17	5.8463E-15	5.8463E-15	2.86
Phosphonate and phosphinate metabolism	6	0.05622	5	3.6102E-10	1.2636E-07	6.336E-08	1.3333
Central carbon metabolism in cancer	71	0.66527	9	1.3904E-08	4.8526E-06	1.6268E-06	0.18966
Glutathione metabolism	59	0.55283	8	5.5255E-08	0.000019229	4.8486E-06	0.64286
Citrate cycle (TCA cycle)	30	0.2811	6	2.5922E-07	0.000089949	0.000018197	0.85714
Cysteine and methionine metabolism	52	0.48724	7	4.2163E-07	0.00014588	0.000024665	0.92105
Choline metabolism in cancer	99	0.92763	8	3.1933E-06	0.0011017	0.00016012	0.41026
Pentose phosphate pathway	31	0.29047	5	0.000008548	0.0029405	0.00037505	1.5769
One carbon pool by folate	39	0.36543	5	0.000027355	0.0093828	0.0010669	1.1778
Arginine biosynthesis	23	0.21551	4	0.000053822	0.018407	0.0018891	0.92308
HIF-1 signaling pathway	110	1.0307	7	0.000065844	0.022453	0.002101	0.23077
Arginine and proline metabolism	50	0.4685	5	0.000093056	0.031639	0.0027219	0.43243

Table S5. ORA driven by metabolites/genes

Joint metabolite/gene set	Total	Expected	Hits	Raw *p*	Holm adjust	FDR	Impact
Central carbon metabolism in cancer	108	1.2434	24	6.54E-25	2.30E-22	<1E-21	0.09734
Glycerophospholipid metabolism	159	1.8306	20	9.97E-16	3.49E-13	<1E-12	1.7909
ABC transporters	184	2.1184	19	2.17E-13	7.58E-11	<1E-10	0
Glutathione metabolism	97	1.1168	14	3.96E-12	1.38E-09	<1E-09	0.85714
One carbon pool by folate	65	0.74836	12	7.20E-12	2.50E-09	<1E-09	1.1839

Cysteine and methionine metabolism	120	1.3816	14	7.62E-11	2.64E-08	<1E-08	0.6789
Arginine and proline metabolism	121	1.3931	13	1.07E-09	3.69E-07	<1E-07	0.42202
Protein digestion and absorption	152	1.75	14	1.82E-09	6.28E-07	<1E-07	0
Citrate cycle (TCA cycle)	50	0.57566	9	4.34E-09	1.49E-06	<1E-06	1.3636
Aminoacyl-tRNA biosynthesis	118	1.3586	12	9.18E-09	3.14E-06	<1E-06	0.12371
Phosphonate and phosphinate metabolism	63	0.72533	9	3.62E-08	0.000012339	<1E-05	0.21053
Choline metabolism in cancer	110	1.2664	11	4.71E-08	0.000016012	<1E-05	0.30189
Alanine, aspartate and glutamate metabolism	65	0.74836	9	4.79E-08	0.000016254	<1E-05	0.78689
Glycine, serine and threonine metabolism	89	1.0247	10	6.48E-08	0.0000219	<1E-05	0.80952
D-Amino acid metabolism	75	0.86349	9	1.71E-07	0.000057685	<1E-05	0
Purine metabolism	229	2.6365	14	3.36E-07	0.00011279	<1E-05	0.52113
Arginine biosynthesis	47	0.54112	7	9.55E-07	0.00031998	<1E-04	0.82051
Glyoxylate and dicarboxylate metabolism	95	1.0938	9	1.32E-06	0.00044163	<1E-04	0.26667
Pyruvate metabolism	79	0.90954	8	3.18E-06	0.0010575	<1E-04	0.85714
Mineral absorption	90	1.0362	8	0.000008497	0.002821	<1E-03	0
HIF-1 signaling pathway	125	1.4391	9	0.000012912	0.0042739	<1E-03	0.21687
Glucagon signaling pathway	133	1.5312	9	0.000021286	0.0070245	<1E-03	0.13253
Folate transport and metabolism	51	0.58717	6	0.000024052	0.0079132	<1E-03	0
Pantothenate and CoA biosynthesis	51	0.58717	6	0.000024052	0.0079132	<1E-03	0.15556
Type II diabetes mellitus	53	0.6102	6	0.000030093	0.0098404	<1E-03	0.29032
Antifolate resistance	58	0.66776	6	0.000050636	0.016507	<1E-03	0.03333
Pyrimidine metabolism	124	1.4276	8	0.000087311	0.028376	<1E-02	0.41667
Taurine and hypotaurine metabolism	41	0.47204	5	0.000099597	0.03227	<1E-02	0.15625
Pentose phosphate pathway	68	0.78289	6	0.00012474	0.04029	<1E-02	0.98485
Glycolysis or Gluconeogenesis	98	1.1283	7	0.00012975	0.041778	<1E-02	0.54688
